# Biostimulants derived from organic urban wastes and biomasses: An innovative approach

**DOI:** 10.3389/fchem.2023.969865

**Published:** 2023-02-10

**Authors:** Alessia Giordana, Mery Malandrino, Alfonso Zambon, Gigliola Lusvardi, Lorenza Operti, Giuseppina Cerrato

**Affiliations:** ^1^ Dipartimento di Chimica, Università degli Studi di Torino, Turin, Italy; ^2^ Dipartimento di Scienze Chimiche e Geologiche, Università degli Studi di Modena e Reggio Emilia, Modena, Italy

**Keywords:** biostimulant, hydroxyapatite, silica, rice husk, humic acid, fulvic acid, nanofertilizer

## Abstract

We used humic and fulvic acids extracted from digestate to formulate nanohybrids with potential applications in agronomy. In order to obtain a synergic co-release of plant-beneficial agents, we functionalized with humic substances two inorganic matrixes: hydroxyapatite (Ca₁₀(PO₄)₆(OH)₂, HP) and silica (SiO₂) nanoparticles (NPs). The former is a potential controlled-release fertilizer of P, and the latter has a beneficial effect on soil and plants. SiO_2_ NPs are obtained from rice husks by a reproducible and fast procedure, but their ability to absorb humic substances is very limited. HP NPs coated with fulvic acid are instead a very promising candidate, based on desorption and dilution studies. The different dissolutions observed for HP NPs coated with fulvic and humic acids could be related to the different interaction mechanisms, as suggested by the FT-IR study.

## 1 Introduction

One of the major challenges for the future is the increasing worldwide demand for food and fuel that comes with a growing global population (National Academies of Sciences et al., 2019). Further intensification of current agricultural practices is unsustainable because they have led to unacceptable levels of environmental degradation (European Environment Agency., 2020). The poor efficiency and overuse of NPK fertilizers lead to losses in energy and water and present critical environmental implications such as eutrophication of surface waters and soil degradation caused by leaching of organic matter and salinization (National Academies of Sciences et al., 2019). Innovative agricultural interventions need to be implemented to safeguard ecosystems, biodiversity, and climate; nanotechnology has been suggested as a promising approach to this end because nanoscale materials can provide time-controlled, target-specific, self-regulated, and multi-functional capabilities (Lowry et al., 2019). Nanomaterials can, for example, deliver fertilizers, pesticides, and herbicides in an ‘on-demand’ manner, thus avoiding repeated applications and reducing adverse effects on plants and the environment (Wang et al., 2016). In particular, nanofertilizers may improve nutrient delivery efficiency and enhance productivity by ensuring gradual release of these nutrients (Verma et al., 2022). Among the strategies proposed, the use of biostimulants offers a potential route to reduce the dependency on fertilizers and pesticides.

Biostimulants are classified as any substance or microorganism, which even if not delivering nutrients, when applied to plants enhance nutritional efficiency, abiotic stress tolerance, and/or crop quality traits (du Jardin, 2015). Among the various biostimulants, an important class of compounds is represented by humic substances. Their role in soil fertility is well known, and the stimulation of plant growth by humic substances is well documented in the literature (Calvo et al., 2014; Canellas et al., 2015). Nowadays, commercial products are obtained from non-renewable sources such as leonardite, peat, and coal. The aim of this work is thus to valorize the residual product of a transformation chain of the organic fraction of the solid urban waste, a basic extract of a solid digestate rich in humic and fulvic acids (indicated as HAs and FAs, respectively), which may represent a renewable source of humic substances. It is impossible to define a molecular formula for these heterogeneous and complex molecular systems, whose chemical composition varies depending on the source and generation condition. Humic substances are classified according to their solubility as a function of pH change. FAs represent the fraction always soluble, while HAs represent the fraction insoluble at a pH < 2. Moreover, all these humic substances contain many oxygen-based functional groups (such as phenol and carboxylic acid) that allow their adsorption on an inorganic substrate (Vekariya et al., 2016). By selecting proper solids, it is possible to obtain multi-functional and non-toxic nanocomposites that could find applications in agronomy being capable of conducting multiple beneficial actions including crop nutrition, plant stimulation, and soil amendments.

As inorganic substrates, we choose hydroxyapatite (Ca₁₀(PO₄)₆(OH)₂, HP) and silica (SiO₂) nanoparticles (NPs): the former is a potential solid fertilizer of P and the latter has beneficial effects on soil and plants. Even though Si is not considered an essential element, the beneficial effects of Si on plants and soil have been demonstrated by several studies using pot, hydroponic, and field experiments (Guntzer et al., 2012).

HP is a biocompatible and biodegradable material rich in P. Given its high solubility under acidic conditions and thus in gastric juices, HP does not raise concerns over bioaccumulation through the food chain and negative effects on human health. HP solubility decreases with the increasing pH value, allowing a slow release of nutrients at soil pH. HP NPs have been proposed as a controlled-release fertilizer of P, but their use is still limited due to low solubility in neutral and alkaline soils and their tendency to agglomerate. A strategy proposed to improve dissolution and bioavailability of HP NPs is to modify their surface with plant- or soil-friendly materials. Surface engineering with urea afforded nanohybrid fertilizers with high contents of N (Kottegoda et al., 2017; Sharma et al., 2022) with synergic effects on nutrient use efficiency, decreasing urea solubility and increasing HP dissolution. Surface modification with citric acid has been proven to modulate P-realizing kinetics, providing the optimal concentration of P for crops during their growth (Samavini et al., 2018). Humic acids have been used to modify the surface acidity of HP, in virtue of their ability to stimulate plant growth, to obtain a multi-functional material with multiple beneficial actions (Yoon et al., 2020). In this study, the authors functionalized HP NPs with commercial and synthetized humic substances and tested the resulting materials on *Zea mays*, noting significant improvements in terms of early plant growth, corn productivity, and resistance to abiotic stresses. These effects were ascribed to the co-release of P (as phosphate ions) and humic substances and to their synergic effect as plant-beneficial agents.

As possible oxidic substrates, we employed SiO₂ NPs obtained from rice husks, a common agricultural waste in Piedmont. Si is recognized as fundamental in agriculture, especially when stress conditions are afflicting plants, even though it is not recognized as “essential” for plant growth (Luyckx et al., 2017). Moreover, many widespread crops are Si accumulators (such as rice, wheat, sugarcane, and soybean), and their absorption depends on its availability (Guntzer et al., 2012). Furthermore, various studies have demonstrated the beneficial effect of Si on soil (Aksakal et al., 2013) and suggested that Si can make P in soil more available (Guntzer et al., 2012). Moreover, Si protects plants against various pathogens, as its presence enhances the cell wall resistance to fungal attacks. Rice husks are costless and widely available agricultural wastes, and they contain 15–20 wt% of silica in hydrated amorphous forms, depending on the variety, climate, and geographic location of production (Sun and Gong, 2001; Chandrasekhar et al., 2004). Rice husks represent a safer, less expensive, and more environmentally friendly silica precursor with respect to commonly employed alkoxysilane compounds. On the other hand, Si in the form of SiO₂ might be obtained from rice husks by several different procedures, such as thermal/hydrothermal treatments, chemical attacks, or biological digestion (Shen, 2017; Hossain et al., 2018), leading to a possible re-use of a typical agricultural waste in a “circular economy” approach (rather than dumping or burning it in open spaces), including its use as raw materials in the chemical industry or as an alternative fuel to produce energy (Bakar et al., 2016). Nitric acid digestion permits the extraction of amorphous SiO₂ from rice husks with high purity and interesting properties as the adsorbent of organic dyes (Tolba et al., 2015).

Self-assembly of HAs and FAs on the surface of HP and SiO_2_ NPs was performed by water dipping. NPs coated with HAs and FAs were characterized, and acid desorption and HP dissolution were also evaluated, comparing the effect of FAs and HAs. An FT-IR study was performed to investigate the nature of the interaction between humic substances and the inorganic substrate.

This experimental approach is novel as, to the best of our knowledge, no other studies report the following: (i) the approach to self-assembling humic substances derived from a recycling plant onto inorganic substrates; (ii) the use of silica support derived from waste materials, and (iii) the study of the release of elements/substances (even in a simple medium, like water) from the self-assembled composite(s).

## 2 Materials and methods

### 2.1 Materials

Calcium hydroxide (Ca(OH)₂, 95%) was purchased from Thermo Scientific. Hydrochloric acid (HCl, p.a. ≥37%), ammonia solution (NH₃, p.a. 28%–30%), and nitric acid (HNO₃, p.a. 65%) were purchased from Merck. Phosphoric acid (H₃PO₄; 85%) was purchased from CARLO ERBA. NaHCO₃ was purchased from Fluka. All commercial reagents were used as received without any further purification. The basic extract of the solid digestate containing 7 wt% of HAs and FAs was obtained by a pilot implant treating urban organic wastes (ACEA Pinerolese SpA, Pinerolo (TO), Italy). Rice husks were provided by a local farm (Azienda Agricola Risicola Allocco, Bra (CN), Italy).

### 2.2 Preparation of inorganic substrates

HP NPs were synthesized according to a previously reported precipitation method (Aina et al., 2012). Briefly, 0.1 mol of Ca(OH)₂ was stirred in 200 mL of water for 20 min until a homogeneous suspension was obtained. Then, 0.06 mol of H₃PO₄ (diluted with 200 mL of water) was slowly added dropwise, with continuous stirring. During the reaction, the pH is controlled and maintained above 10.5 by addition of ammonia solution, as necessary. The suspension was stirred for further 2 h and then allowed to stand overnight, and the final product was filtered and dried under ambient conditions. The overall yield is 9.05 g (99.0%).

Rice husk was washed with water to remove the residual of rice and earth. Then, it was dried in an oven at 60°C. Acid digestion was conducted under hydrothermal conditions: rice husk, HNO₃, and pure water taken in a weight ratio of 1:5:5 were allowed to react at 160°C for 2 h. The solid product was separated by filtration under suction, washed with distilled water, and dried under ambient conditions. Using 2.0 g of rice husks, 10 mL of water, and 7.0 mL of HNO₃, 248 mg of SiO₂ was obtained (yield: 12.4 wt%).

### 2.3 Formulation of solid biostimulants

The basic extract was centrifuged (3,000 rpm; 15 min) to eliminate solid residues. Then, HAs and FAs were separated according to different solubility fractions depending on the pH value. In detail, HCl solution (1.0 M) was added to the black dense solution until pH∼1 was achieved, thus allowing the precipitation of a fraction of HAs. The obtained black powder was separated by centrifugation (5,000 rpm; 10 min) and dried in an oven at 55°C. The supernatant solution containing FAs was dried in an oven at 55°C to obtain a solid product.

Solid HAs and FAs were dissolved in either NaHCO₃ solution (0.05 M) or water. To favor a complete dissolution, solutions were sonicated for 5 min and then centrifugated to separate undissolved residues (2,500 rpm, 5 min). Either HP (1.0 g) or SiO₂ (0.5 g) NPs were added to 40 or 20 mL of humic substance solutions and shook for 2 h. The coated NPs were isolated *via* centrifugation (5,000 rpm; 5 min) and dried at 60°C. The amount of adsorbed acids was calculated by the difference in concentration between starting and separated solutions, by the spectrometric detention of absorbance at 254 nm. Calibrations were performed with solutions with known concentrations, according to the Beer–Lambert law. UV-vis spectra were recorded using the Perkin Elmer Lambda 900 spectrophotometer, utilizing quartz cells with an optical path of 1 cm.

### 2.4 Acid and ion release studies

To evaluate the release of humic substances and dissolution of the matrix, biostimulants based on HP (0.26 g) were suspended in 12 mL of pure water. Every 24 h, the solid was separated by centrifugation (5,000 rpm; 5 min), and the same volume of fresh water was re-added. HAs and FAs desorption was quantified by the spectrometric detention on the separated solution, as described previously. The released P and Ca ions were quantified by inductively coupled plasma atomic emission spectroscopy (ICP-AES) (PerkinElmer, model Optima 7000 DV). The wavelengths were 213.617 and 317.933 nm for P and Ca, respectively. Calibrations were performed with standard solutions. The concentration of Ca and P on the used coated HP was determined in the same way by diluting in water the solution obtained after digestion of 0.24 mg of each sample in aqua regia.

### 2.5 Instrumental characterization

Identification and characterization of crystalline phases of pure and coated inorganic substrates were carried out by several solid-state techniques.

PXRD, using a X’Pert powder diffractometer operating in the Bragg–Brentano geometry, equipped with a graphite crystal monochromator, and using Cu(Kα1) radiation (*λ* = 1.5406 Å), was collected in the range of 5°–70° with a step size of 0.02°.

FT-ATR (Attenuated Total Reflection) spectra were obtained in solid samples using the Bruker VERTEX 70 spectrophotometer equipped with Harrick MVP2 ATR cells and a DTGS detector (64 scan, 4 cm⁻^1^ resolution).

Thermogravimetric analyses were carried out on TA Instruments (TGA 2950 hr) in the air flow, with the ramp rate 10°C/min, from rt to 750°C.

The general morphology/topography of various samples was assessed by means of a scanning electron microscope operating with a field emission source (model: TESCAN S9000G; source: Schottky type FEG; resolution: 0.7 nm at 15 keV (in In-Beam SE mode), fitted with an EDS detector (Oxford EDS Ultim Max operating with Aztec software). Before the investigations, all samples were coated with Au in order to enhance the conductivity.

Analyses relative to the ultimate morphology of the samples were performed on a HR-TEM JEOL 3010-UHR (Tokyo, Japan) high-resolution transmission electron microscope (acceleration potential: 300 kV, LaB₆ filament) fitted with an Oxford INCA X-ray energy-dispersive spectrometer (X-EDS) with a PentaFET Si(Li) detector. The samples were dry dispersed before the investigation on Cu grids covered with lacey carbon without any further treatment.

Specific surface areas were evaluated from N_2_ adsorption/desorption isotherms under subcritical conditions (196°C), obtained by means of an ASAP 2020 instrument. The surface area was calculated using the B.E.T. model.

FT-IR spectra were recorded using a Bruker Equinox 55 spectrometer, equipped with an MCT detector at 4 cm⁻^1^ resolution. The solid samples, in the form of self-supported pellets (≈10 mg/cm^2^), were inserted into a conventional quartz vacuum cell equipped with KBr windows connected to a glass vacuum line (residual pressure <10^−5^ Torr) that allows the performance of *in situ* adsorption/desorption runs. The samples were activated at 120°C. Adsorption/desorption tests of water were carried out at 25°C.

## 3 Results

### 3.1 Characterization of inorganic substrates

**FIGURE 1:**
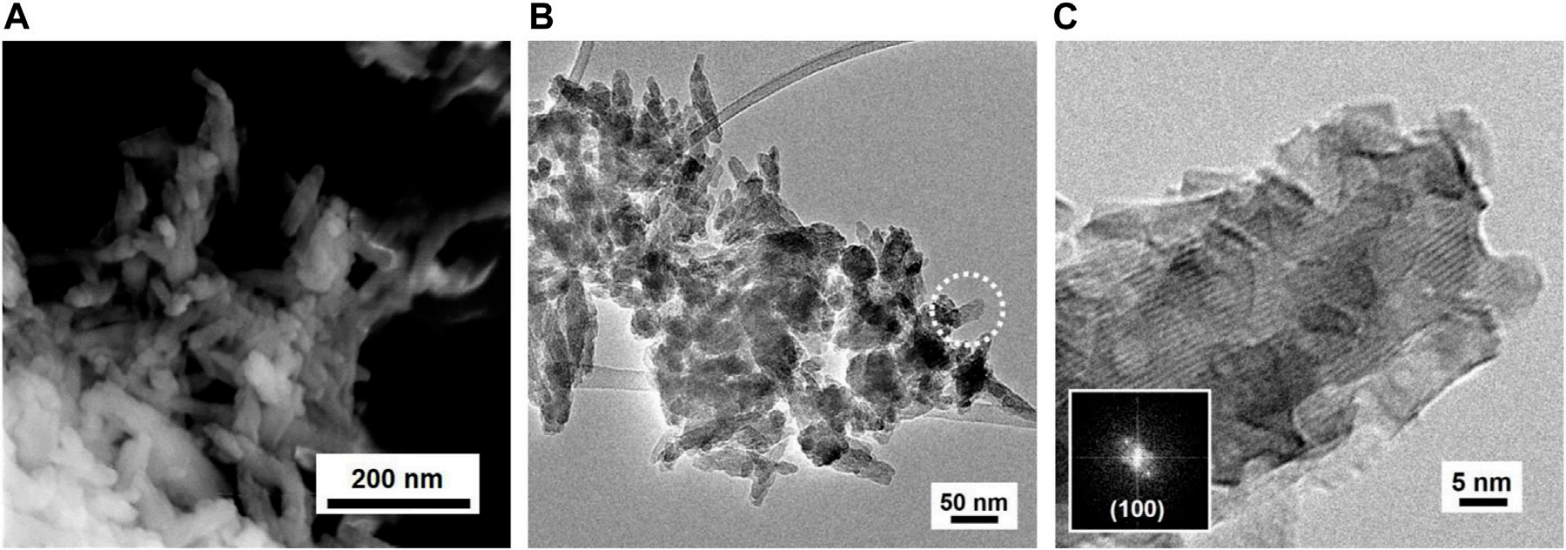
SEM **(A)** and TEM **(B, C)** images of the obtained HP NPs.

The synthetized HP NPs exhibit physico-chemical characteristics similar to those reported in the literature (Aina et al., 2012). The PXRD pattern indicates the formation of a crystalline product, and both peak positions and relative intensities are the characteristics of the HP pure crystalline phase (ICDD card PDF n. 01-074-0565), as reported in Supplementary Material S1 (Supplementary Figure S1). All peaks are broad, suggesting the formation of NPs. In the FT-ATR spectrum (see Supplementary Figure S2), it is possible to recognize intense bands originating from PO₄³⁻ groups (Koutsopoulos, 2002). The most intense and broad band with the maximum at 1,024 cm⁻^1^ is assigned to the asymmetric stretching mode (ν_3_) of the P–O bond, whereas the sharp signals at 627, 601, and 560 cm⁻^1^ are ascribable to the bending modes (ν_4_) of the O–P–O group and the weak component at 963 cm⁻^1^ is assigned to the symmetric mode (ν_1_) of P–O. The spectral component typical of free hydroxyl groups is covered by the presence of a broad band centered at 3,400 cm⁻^1^, due to the stretching mode of water molecules/OH groups interacting via H-bonding, either in bulk or adsorbed at the surface: their relevant bending mode is observable at 1,640 cm⁻^1^ (Sakhno et al., 2015). Three other spectral components are also well evident: a shoulder, located at 875 cm⁻^1^, corresponding to the out-of-plane bending mode (ν_2_) and multiple bands at 1,420, 1,455, and 1,487 cm⁻^1^, attributable to the asymmetric stretching mode (ν_3_), all of them being characteristic of surface carbonate species (Ren et al., 2014). The FESEM image (Figure 1A) indicates the formation of aggregates of prismatic NPs with average dimensions below 200 nm, mostly having a needle-like shape. NPs seem to be homogeneous in shape and dimension, as also confirmed by TEM images (Figures 1B, C and Supplementary Figure S3). As shown in Figure 1C, HP consists of prismatic elongated NPs grown along a crystallographic direction, mainly referred to that of the (101) planes. The SSA is calculated as 122 m^2^/g.

**FIGURE 2:**
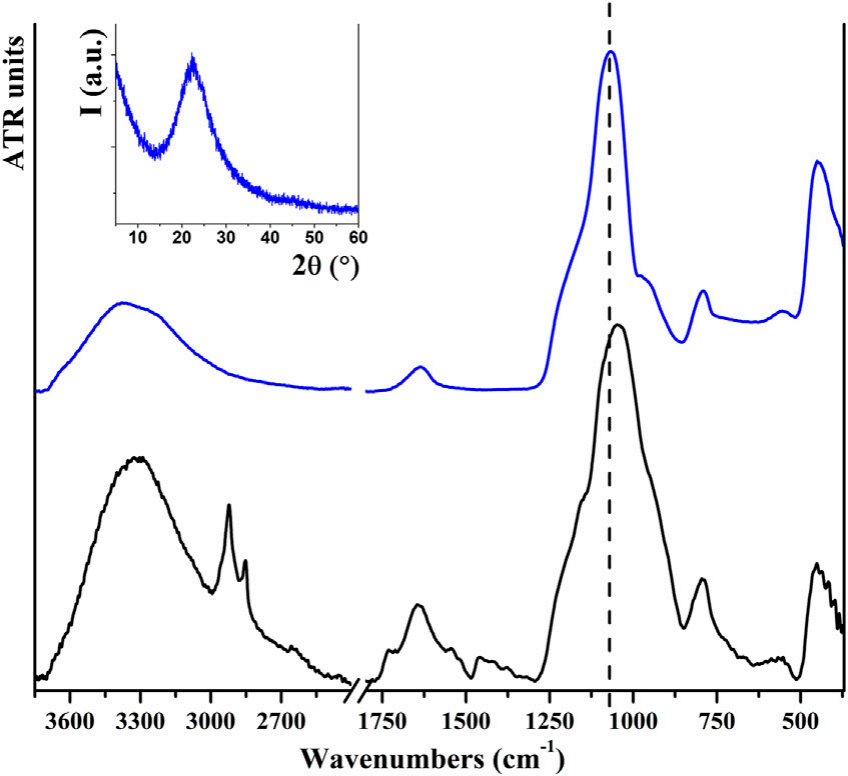
FT-ATR spectra of rice husks (black) and the product obtained after acid digestion (blue). Inset shows the PXRD pattern of the obtained SiO_2_ NPs.

The white powder obtained from the acid digestion of rice husks consists of pure SiO₂ NPs. The hydrothermal acid digestion was repeated different times, always affording analogous products and similar yields. TGA was used to verify the purity of the product. In the thermal degradation of rice husks (in Supplementary Figure S4), a weight loss of 5.2% was observed below 100°C that can be related to the loss of residual water. The majority of the weight loss (78.6%) takes place between 220°C and 500°C and can be related to the decomposition of organic molecules, such as lignin and cellulose. The residual weight at 750°C is 12.8%, very similar to that of the acid digestion synthetic yield. The residual white product collected after thermal degradation of rice husks was also analyzed by FT-ATR and resulted to be composed of SiO₂, as the main components can easily be ascribed to those of pure SiO₂ (Bellamy, 1968; Legrand, 1999). Conversely, the main weight loss of the white powder obtained after acid digestion is very low (4%) and is related to water removal at around 100°C, while a very low loss (0.4%) is observed at 310°C and can be related to the decomposition of the organic residue. Vibrational data obtained for this sample (reported in Figure 2) confirm the formation of SiO₂, presenting strong signals at 1,065, 793, and 450 cm⁻^1^ that represent characteristic modes related to the presence of Si−O−Si bonds. It is also possible to observe two other bands, located at 3400 e 1630 cm^−1^, respectively, related to water stretching and scissor modes, but no signals of organic residue are detectable, confirming the TGA results. The characteristic signals of rice husks, as stretching modes of the C–H bond (around 2,900 cm⁻^1^) and C−C/C−O bond (around 1,000 cm⁻^1^), are not present in the spectrum of the product. The intense signal at 1,050 cm⁻^1^ observed for the rice husk could be due to the superimposition of C−O and Si−O−Si modes, and in the final product we observe a shift at 15 cm⁻^1^. PXRD analysis indicates that the obtained material is amorphous, as evident from the inset to Figure 2. FESEM images indicate the presence of aggregates of small NPs that maintain an aggregation similar to that observed in the starting material (Supplementary Figure S5). The SSA is calculated as 202 m^2^/g.

### 3.2 Adsorption of humic substances on inorganic matrixes

The solid fractions obtained by the separation of the basic extract were characterized by FT-ATR spectroscopy (see Supplementary Figure S6). HAs and FAs present similar functional groups (phenolic and alcoholic hydroxyl groups, aromatic and aliphatic carboxyl species and carbonyl groups, aliphatic chains and amides) and give then very similar FT-IR spectra but with some differences in the relative content of these groups (supported semi-quantitatively by the intensities of the relevant spectral components), and a careful analysis can reveal specific characteristics of the two fractions. Spectra are dominated by a broad band in the range of 3,700–2,500 cm⁻^1^, ascribable to the stretching mode of hydroxyl groups of carboxyl species and phenolic and alcoholic functional groups interacting via H-bonding. For FAs, this component is very intense and exhibits a shoulder at 3,240 cm⁻^1^ that could be attributable to N–H aminic stretching vibrations. Vibrational features of HAs are represented by a couple of bands located at 2,925 and 2,855 cm⁻^1^, ascribable to the stretching mode of aliphatic C−H in methyl (−CH₃) and methylenic (−CH₂−) units, respectively, which are superimposed to the OH stretching band and more evident with respect to FAs, consistent with the literature (Giovanela et al., 2004). The higher concentration of carboxylic groups in FAs can be related to the higher intensity of the bands lying above 1,600 cm⁻^1^ (Giovanela et al., 2004; Tatzber et al., 2007). Finally, the broad signal at 1,210 cm⁻^1^ is ascribable to the C−O stretching mode of alcoholic and phenolic groups, and it is more intense for HAs. An additional band is evident at 1,320 cm⁻^1^, which is most likely due to bending modes of aromatic amine and, in fact, it is more intense for FAs.

**FIGURE 3:**
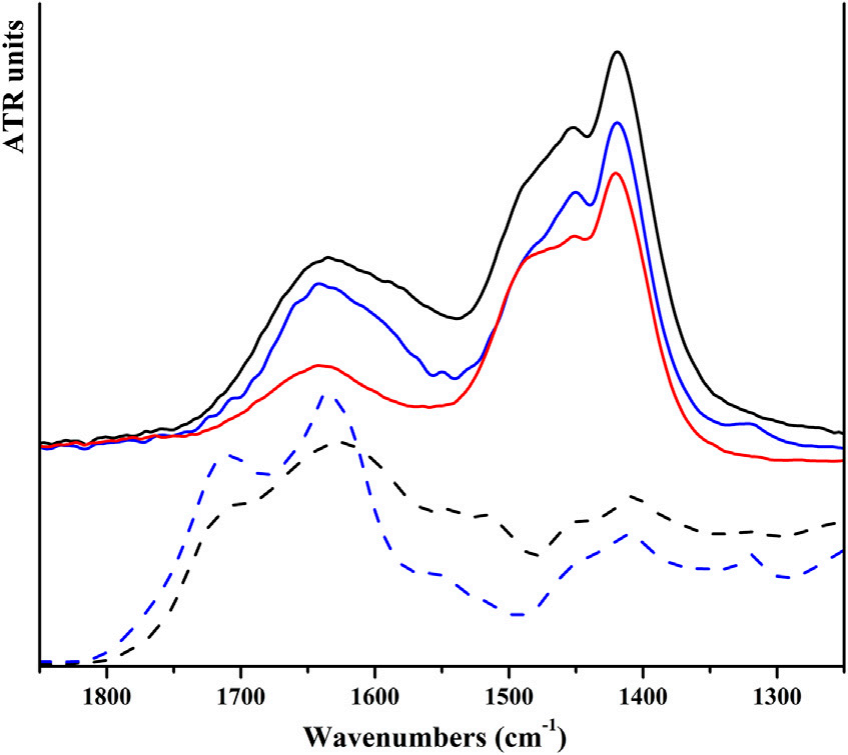
FT-ATR spectra of HP NPs (solid red line), solid HAs (black dash line), solid FAs (blue dash line), and HP NPs coated with HAs (solid black line) and FAs (solid blue line) in the spectral range of 1,250–1,850 cm⁻^1^.

**FIGURE 4:**
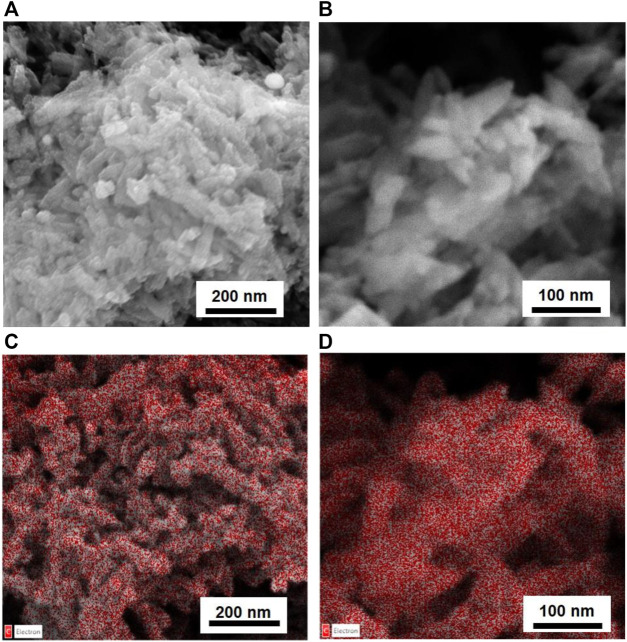
SEM images of HP NPs coated with HAs **(A)** and FAs **(B)** and EDS carbon maps **(C)** for HAs and **(D)** FAs.

NPs of both products were dipped in HAs and FAs solutions, having a concentration near saturation. To dissolve HAs, it was necessary to use a NaHCO_3_ solution (0.05 M); the pH of the final solution is 8.5. Of note, commercial HA salts also dissolve giving a basic solution. Instead, FAs were dissolved in water, and the final solutions have a pH of 4.5. For HP NPs, the reciprocal affinity of the two materials resulted in a fast functionalization, as evident by (i) a drastic color change of the solid from white to brown and (ii) the discoloration of the supernatant solution. Conversely, on SiO_2_ NPs, no evident chromogenic features of humic substances are observed. UV-vis analysis of supernatant solutions was used to determine the amount of acids adsorbed on all solids. For both inorganic substrates, a higher amount of FAs is absorbed with respect to HAs, as shown in Table 1. We can observe that HP is able to absorb more humic substances from solutions with respect to silica and that humic substances are absorbed on each substrate in similar amounts, independent of the kind of acid (average value: 32.5 for HP and 12.9 for SiO_2_). The amount of adsorbed acids is very low for silica-based materials, and no evidence of functionalization is observable in the vibrational spectra and by means of other characterization techniques. Meanwhile, for HP-based materials, FT-ATR spectra present typical features of HP and humic substances; this is better visible in Figure 3, in which a magnified view in the 1,250–1,850 cm⁻^1^ range has been reported. Within this region, the spectrum of HP is featured by the spectral component ascribable to water and carbonate species, whereas the spectra of HAs and FAs are characterized by broad bands above 1,600 cm⁻^1^, ascribable to COO^−^ and aromatic groups (as discussed previously). The bands of HAs and FAs were superimposed to those of the inorganic substrate, but in the spectra of coated materials, a broad shoulder at around 1,590 cm⁻^1^ is observable. PXRD patterns indicate that the coated HP NPs retain a higher degree of crystallinity, without any peculiar phase modification (Supplementary Figure S7). FESEM images suggest that both morphology and dimensions are maintained after the functionalization step (see Figure 4). EDS maps of carbon provide evidence that a higher density of organic molecules is observed for FAs derivatives that seems to form a coated film on top of the HP crystallites.

**TABLE 1:** Amount of HAs and FAs adsorbed on inorganic matrixes.

	wt% of acids on the substrate	mg of absorbed acids	Concentration of the initial solution (mg/mL)	wt% of acids adsorbed from the initial solution
HP + HAs	5.37	26.8 ± 1.5	4.59 ± 0.08	29.2
SiO_2_ + HAs	3.28	16.4 ± 0.2	5.86 ± 0.08	14.0
HP + FAs	13.4	67.2 ± 2.2	9.38 ± 0.11	35.8
SiO_2_ + FAs	5.23	26.1 ± 23.4	11.2 ± 01.2	11.7

### 3.3 Co-release of ions and humic substances in water

**FIGURE 5:**
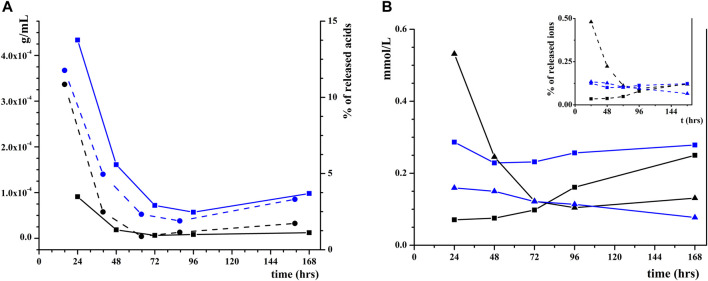
**(A)** Concentrations of HAs (black squares) and FAs (blue squares) released in pure water and wt% of the released HAs (black circles) and FAs (blue circles); **(B)** Concentrations and wt% (in the inset) of solubilized P (triangles) and Ca (squares) from HP NPs coated with HAs (black) or FAs (blue).

To understand if there are differences in HP dissolution depending on the nature of adsorbed organic acids (HAs or FAs), a specific test was performed (as reported in Section 2.4), aimed at quantifying the concentration of relased acids and revealiong the amount of phosphate and calcium ions released in water by coated HP (in steps of 24 h), as shown in Figure 5 and Supplementary Material S1 (Supplementary Table S1; Supplementary Table S2). Results indicate that for both HAs and FAs, a maximum concentration is reached after the first 24 h (9.1 and 4.3 mg/mL, respectively), but in the successive steps, the release is almost constant (see the solid line in Figure 5A). The difference in concentration can be related to the different amount of adsorbed acids on HP, but comparing the percentage of released acids (see the dash line in Figure 5A) a very similar trend between HAs and FAs is observed. The same is not true for HP dissolution, as shown in Figure 5B. In fact, for HP coated with FAs, the concentration of both P and Ca species in water is virtually constant during the week (average values are 0.12 and 0.26 mmol/L, respectively). On the contrary, for the material coated with HAs, the concentration of P is very high after the first 24 h (0.53 mmol/L) and then it drastically decreases (0.25 mmol/L after 48 h down to 0.12 mmol/L after 72 h), whereas the concentration of Ca slowly increases during the week (starting from 0.071 mmol/L after 24 h and reaching 0.16 mmol/L after 96 h). The same trend is visible when observing the percentage in weight of released Ca and P (in the inset in Figure 5B), suggesting that dissolution is influenced by the kind of acid adsorbed on the surface. Comparing the total amount of ions present in the solution, a higher amount of HP is dissolved when the surface is coated with FA (0.85 mg, 0.23 of P and 0.62 of Ca) than HA (0.73 mg, 0.42 of P and 0.31 of Ca).

### 3.4 FT-IR study of the interaction mechanism

**FIGURE 6:**
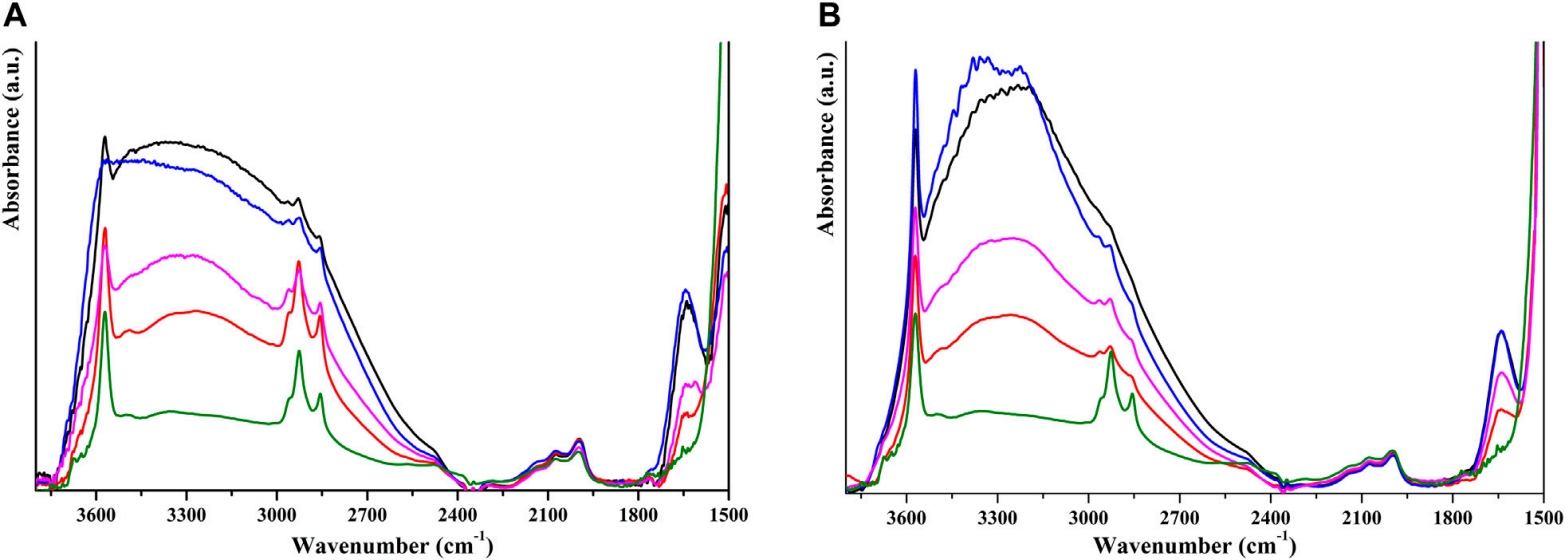
FT-IR spectra of self-supported pellets of HP NPs coated with FAs **(A)** and HAs **(B)** after outgassing (black lines), thermal activation at 120°C (red lines), addition of water vapor (blue lines) and outgassing (pink lines). Spectra of starting HP NPs after thermal activation at 120°C are reported as green line.

A FT-IR study was performed to investigate the nature of the interaction between HP and humic substances. For the system coated with FAs, in the spectrum of the sample after outgassing (black line in Figure 6A), it is possible to recognize the signals of HP, particularly the overtones of the phosphate group (between 1,950 and 2,200 cm⁻^1^) and the stretching mode of surface-isolated hydroxyl species at 3,570 cm⁻^1^. It is also possible to recognize the stretching modes of aliphatic moieties of FAs around 2,925 cm⁻^1^ and the stretching mode of carboxylic groups around 1,650 cm⁻^1^. These signals are superimposed to the typical HP band. We can observe that the signals of C–H stretching are more intense in the coated materials, suggesting that this is in part related to the presence of FAs. The band of physisorbed water can be removed by thermal treatment at 120°C, and comparing coated and pristine HP (red and green lines in Figure 6A, respectively), we can attribute these signals to FAs. We can hypothesize that FAs and HP mainly interact through H-bonding of carboxylate and hydroxyl groups with phosphate and hydroxyl surface groups, as supported by the presence of a signal at 3,490 cm⁻^1^ that can be assigned to columnar OH groups interacting *via* H-bonding with the carboxylate group (Bertinetti et al., 2007) and by the broad band centered at 3,300 cm⁻^1^, related to OH groups interacting *via* H-bonding. After water re-addition, the disappearance of stretching modes of the isolated OH surface group is observed (blue line in Figure 6A), suggesting that part of these surface groups is not involved in the interaction with FAs and is still free to interact with water molecules. This interaction with water is weak as a simple outgassing at room temperature brings about its removal. When the same experiment is carried out on HP coated with HAs, it can be noted that the signal of isolated OH groups is more evident in every step and that the interaction with water is also hindered at high water pressure, as shown in Figure 6B. The bands around 2,900 cm⁻^1^ are less evident, and this can be related to the low percentage of HAs on the HP surface. The signal at 3,490 cm⁻^1^ is less evident with respect to HP coated with FAs, suggesting that in this system the interaction *via* H-bonding is less important and part of HAs may interact by Lewis acid–basic interaction with Ca ions, as reflected in different behaviors observed during the dissolution of coated HP.

We also performed the same experiment on coated SiO_2_, but no signals ascribable to humic substances can be observed, probably because of their low amount. Thermal activation at 120°C is not sufficient to remove physisorbed water, but a higher temperature treatment would cause HAs and FAs degradation, so no indication about the substrate–acid interaction has been obtained for these systems.

## 4 Discussion

Crystalline HP NPs were obtained by aqueous precipitation and coating with HAs and FAs, obtained from the valorization of urban organic waste. HP coated with our HAs presents characteristics similar to those reported for HP coated with commercial HAs (Yoon et al., 2020). A higher amount of FAs is adsorbed on the HP surface with respect to HAs (13 vs*.* 5.4 wt%, respectively). FAs and HAs present similar functional groups, but in model structures FAs have less condensed aromatic rings, a higher fraction of aliphatic chains, and a higher density of carboxylic acid groups (Aiken, 1985). Analysis of spectroscopical data on FAs and HAs obtained from organic waste also indicate the same trend in our samples, suggesting a similarity with soil humic substances (Giovanela et al., 2004; Tatzber et al., 2007). It is known that humic substances have a tendency to agglomerate depending on pH conditions (de Melo et al., 2016), and it is important to note the different pH values of HAs and FAs solutions in the adsorption step. To re-dissolve solid HAs, it is necessary to basify the solution till pH reaches around 8.5. In the basic environment, phenolic and carboxylic groups are deprotonated, and the repulsion of these negatively charged groups causes molecules to assume a stretched configuration. We can presume that this structure is maintained after adsorption, preventing free hydroxyl groups to interact with water during the FT-IR experiment, possibly due to steric reasons. The solution of FAs instead has a pH of around 4.5, and in the acid condition, most phenolic and carboxylic groups are protonated, the repulsion is minimized, and the molecules adopt a coiled and compact structure. These can also explain the different amount of adsorbed species because folded molecules can be absorbed in higher quantity with respect to those having a stretched configuration.

HAs and FAs present a similar behavior in desorption from HP, while the nature of the acid influences the dissolution mechanism of coated HP. HP dissolution is more effective for FAs systems, where it is constant with time and more concentrated in Ca. For HA systems, the release is less constant, and after the first step (24 h) the concentration of P released in the solution drastically decreases, while the concentration of Ca increases with time. In a recent study on the dissolution of HP obtained from fish bones, similar values of P dissolution were obtained suspending the material in a citric acid solution (Sittitut et al., 2022), suggesting that surface coverage with HAs and FAs generates a local acid environment in the presence of water that favors HP dissolution. Our results suggest that HP NPs coated with FAs is a potential material for a synergic release of crop nutrients and stimulants, presenting a more controlled and efficient dissolution with respect to HP coated with HAs.

HP coated with FAs and HAs has shown a different behavior in the adsorption step and a different effect on HP dissolution. These could be correlated to a different interaction mechanism between the substrate and organic acids. The literature suggests that oxygen-based functional groups on the surface of HP nanoparticles can bind with two competing mechanisms, i.e., H-bonding interaction or chelation of Ca^2+^ ions (de Melo et al., 2016; Yoon et al., 2020). FT-IR studies (see Figure 6) suggest that the competition between interaction mechanisms is more pronounced on HP coated with HAs, in which a large fraction of OH groups remains free from interactions; conversely, the H-bonding interaction seems to be predominant when the material is coated with FAs. As this is a stronger interaction, we could suppose that desorption of FAs and HAs interacting *via* H-bonding promotes HP dissolution more than the desorption of HAs, which interact with Ca^2+^, as also suggested by the desorption study.

Finally, we have shown that it is possible to obtain pure SiO_2_ NPs from rice husks in a more sustainable way with respect to other previously reported procedures (Sun and Gong, 2001; Hossain et al., 2018) using water as a solvent and without any combustion treatment. This hydrothermal digestion procedure is fast, reproducible, and allows to obtain amorphous NPs with a high surface area (202 m^2^/g). Despite the material characteristics being promising as humic substance adsorbers, having a high SSA and a certain amount of surface hydroxyl groups indicate that a very low amount of humic species can be really adsorbed. Due to the interesting properties of SiO_2_ obtained from rice husks, further studies will be performed in order to investigate the possible functionalization of the surface to obtain a catalyst from a worldwide common agricultural waste.

Some preliminary conclusions drawn regarding this study are as follows: first, we demonstrated that it is possible to obtain a composite material (by self-assembly) to be employed in a gradual delivery process starting from waste materials: in our opinion, this is a result that might boost the interest towards such applications, particularly for circular economy applications and processes. Second, the physico-chemical characterization (mainly carried out by means of vibrational spectroscopy but not restricted to this) demonstrated that the different dissolution behaviors exhibited by the composite materials might be due to the different interaction mechanisms that regulate the adsorption/desorption of humic substances to/from the inorganic supports. Finally, the opportunity of obtaining silica NPs from another waste product (from agriculture) is in our opinion a very promising approach that deserves further investigations to shed some more light onto the general valorization process of wastes.

## Data Availability

The original contributions presented in the study are included in the article/Supplementary Material; further inquiries can be directed to the corresponding author.
