# Supplementary material for: Biostimulants derived from organic urban wastes and biomasses: An innovative approach
Source: Front Chem. 2023 Feb 10;11:969865. doi: 10.3389/fchem.2023.969865 (PMC9950392; doi:10.3389/fchem.2023.969865)
Supplement: Supplementary file 1 [file DataSheet1.docx]

Supplementary Material

**
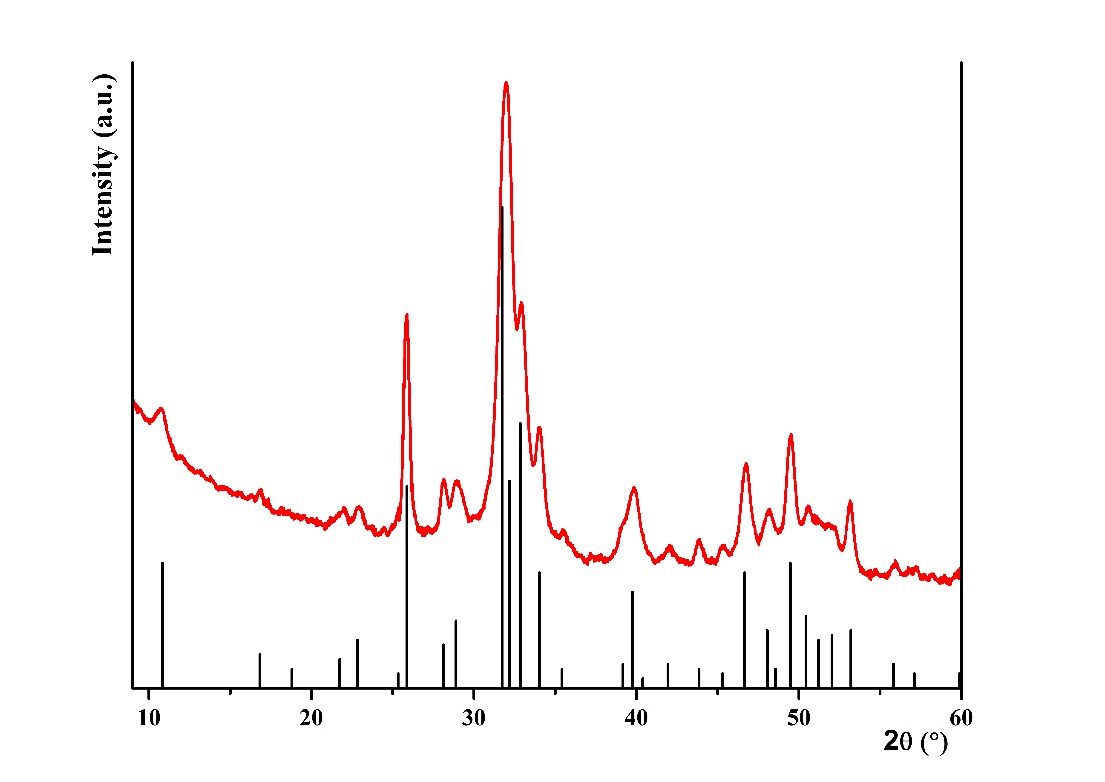
**

**Supplementary Figure 1.** PXRD pattern of HP NPs (red line) compared to the reference (HP, PDF 01-074-0565).

**
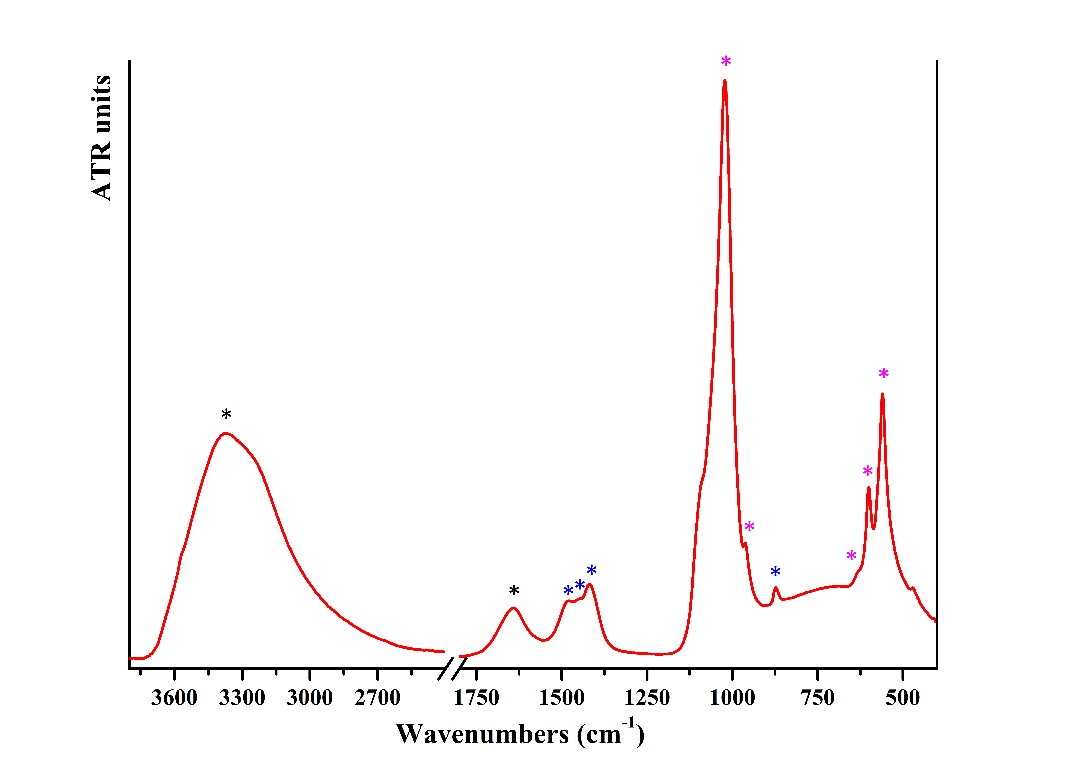
**

**Supplementary Figure 2.** FT-ATR spectrum of HP NPs, star colors are related to band attribution: phosphate groups (pink), water (black), carbonate groups (blue).

1.
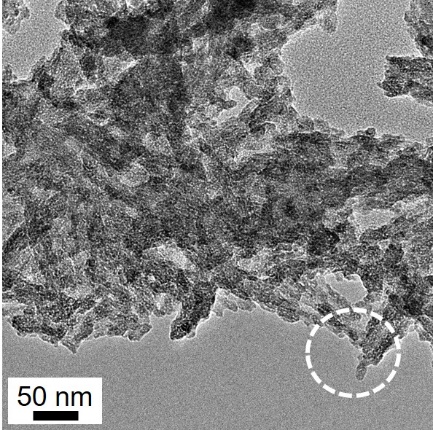
 **(B)**
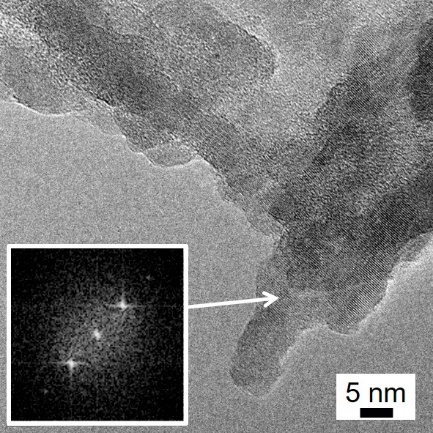
 **(C)**
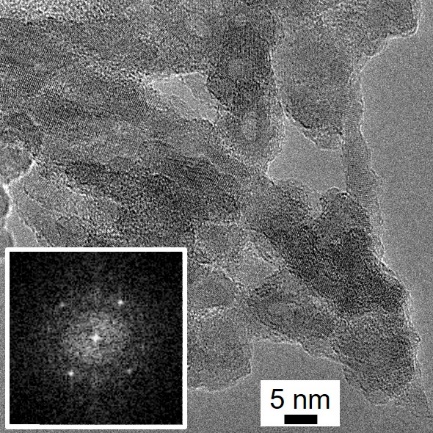


**Supplementary Figure 3.** HR-TEM images of plain HP NPs: general overview (section A), high magnification portions with evidence of fringe patterns and no hypothetical beam damage (section B) and with hypothetical beam damage (if any, section C).

**
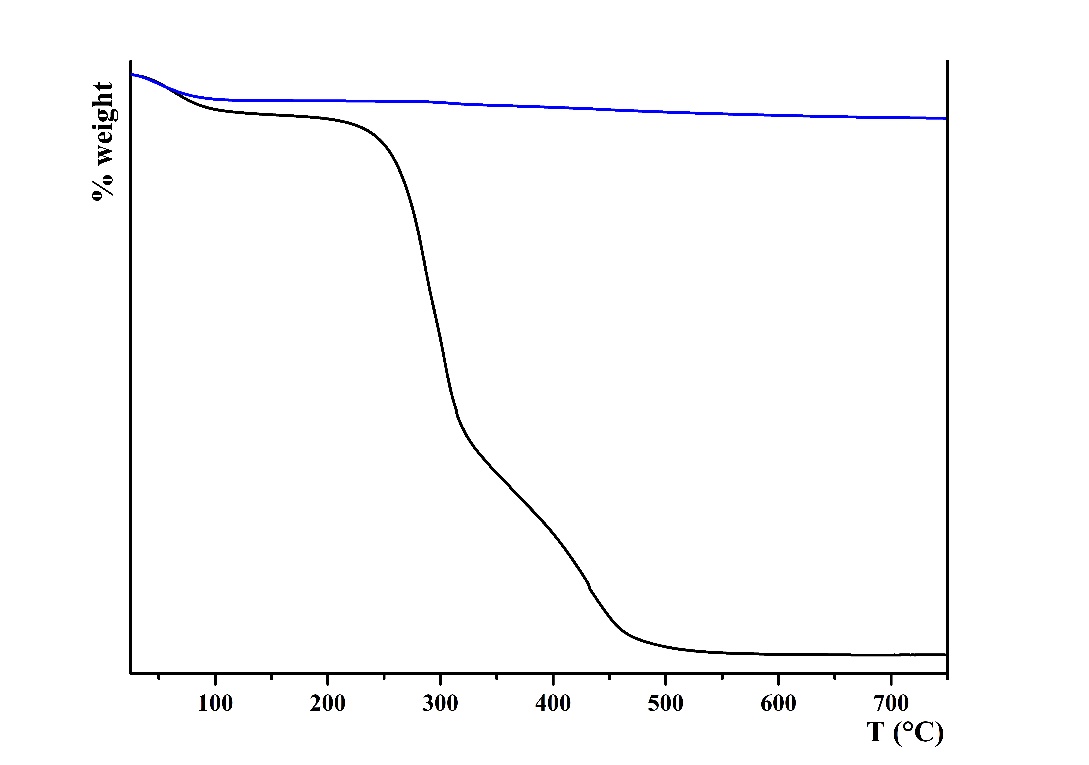
**

**Supplementary Figure 4.** Thermo-gravimetric profile of rice husks (black) and of the derived product (blue).

**(A)** **
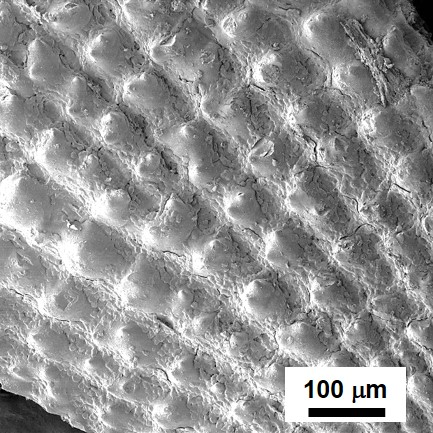
 (B)**
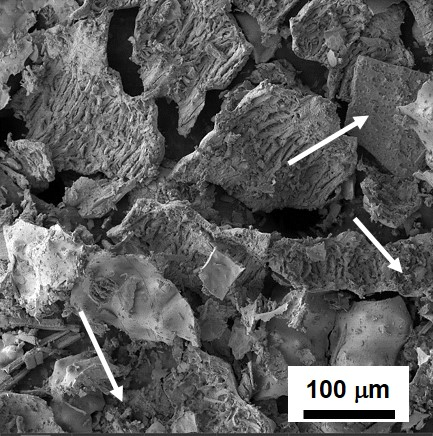


**Supplementary Figure 5.** SEM images of rice husk (a) and SiO_2_ NPs (b) obtained after acid digestion.

**
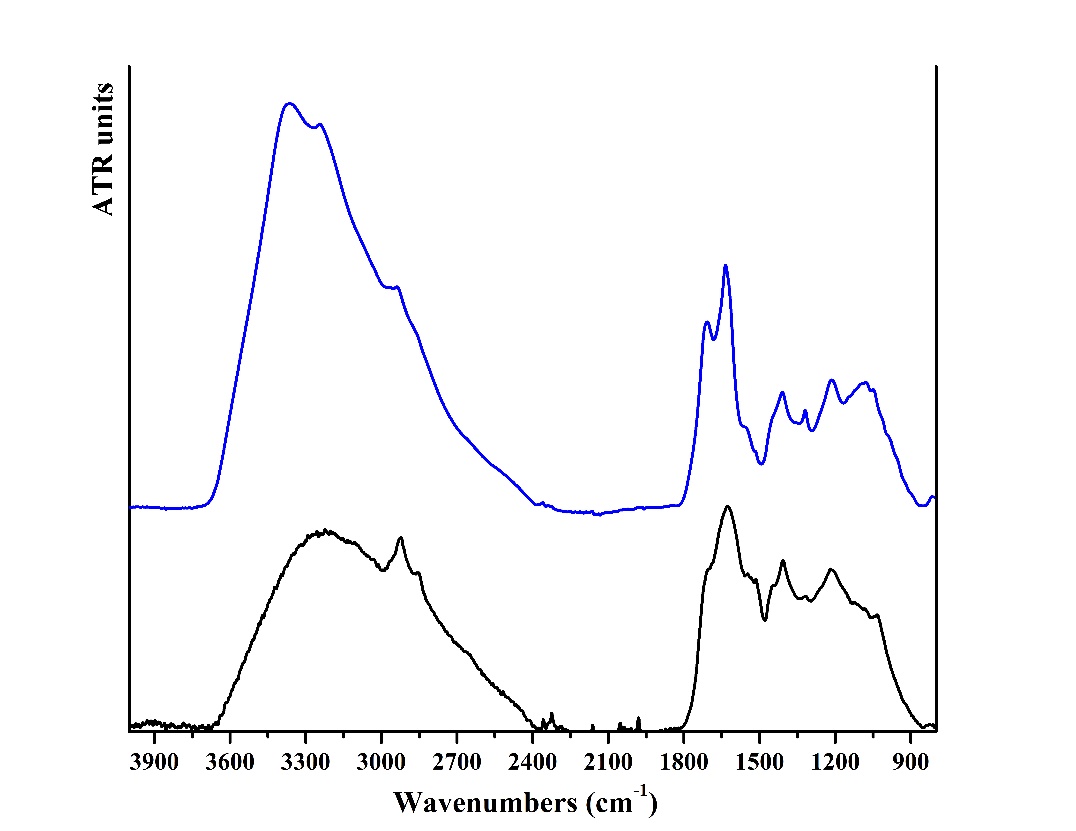
**

**Supplementary Figure 6.** FT-ATR of HAs (black) and FAs (blue).

**
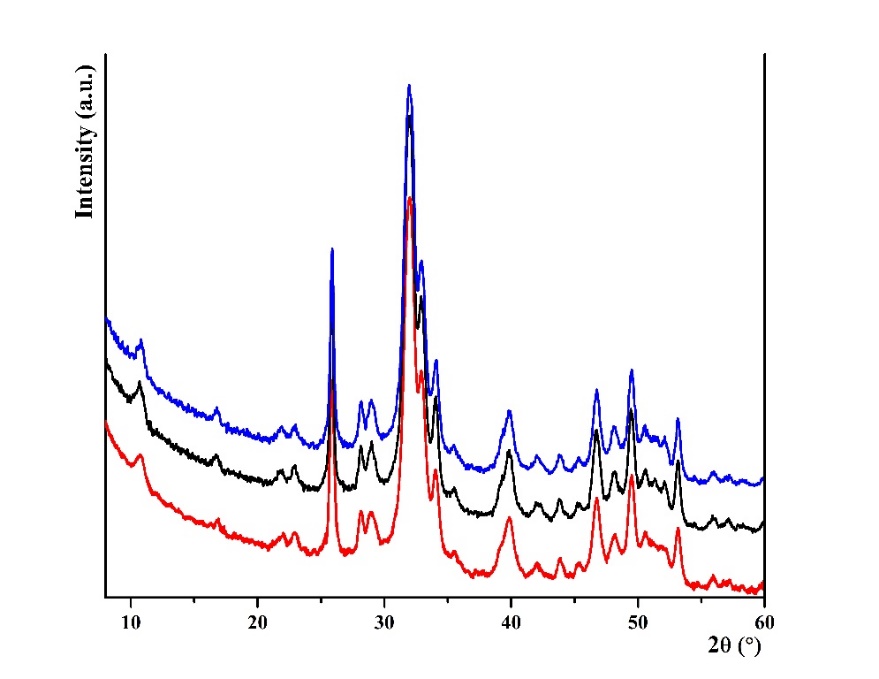
**

**Supplementary Figure 7.** Comparison of patterns of HP NPs (red) and HP NPs coated with HAs (black) or FAs (blue).

**Supplementary Table 1.** Quantities of humic acids, calcium and phosphorous released in water from HP coated with HAs.

| Time (h) | HAs | | Ca | | P | |
| --- | --- | --- | --- | --- | --- | --- |
|  | mg | Released  /total | mg | Released  /total | mg | Released  /total |
| 24 | 1.092 ± 0.009 | 11% | 0.034 ± 0.004 | 0.035% | 0.198 ± 0.003 | 0.48% |
| 48 | 0.223 ± 0.006 | 2.5% | 0.036 ± 0.004 | 0.037% | 0.091 ± 0.003 | 0.22% |
| 72 | 0.076 ± 0.009 | 0.87% | 0.047 ± 0.004 | 0.048% | 0.045 ± 0.003 | 0.11% |
| 96 | 0.099 ± 0.009 | 1.2% | 0.077 ± 0.004 | 0.079% | 0.038 ± 0.003 | 0.095% |
| 168 | 0.148 ± 0.009 | 1.7% | 0.120 ± 0.004 | 0.123% | 0.048 ± 0.003 | 0.12% |
| Total | 1.64 | 17% | 0.315 | 0.322% | 0.422 | 1.03% |

**Supplementary Table 2.** Quantities of fulvic acids, calcium and phosphorous released in water from HP coated with FAs.

| Time (h) | mAs | | Ca | | P | |
| --- | --- | --- | --- | --- | --- | --- |
|  | mg | Released  /total | mg | Released  /total | mg | Released  /total |
| 24 | 5.206 ± 0.011 | 11% | 0.138 ± 0.004 | 0.13% | 0,059 ± 0.003 | 0.13% |
| 48 | 1.933 ± 0.014 | 5.0% | 0.110 ± 0.004 | 0.10% | 0.056 ± 0.003 | 0.13% |
| 72 | 0.864 ± 0.014 | 2.3% | 0.111 ± 0.004 | 0.10% | 0.045 ± 0.003 | 0.10% |
| 96 | 0.685 ± 0.014 | 1.9% | 0.123 ± 0.004 | 0.11% | 0.042 ± 0.003 | 0.096% |
| 168 | 1.177 ± 0.014 | 3.3% | 0.134 ± 0.004 | 0.12% | 0.029 ± 0.003 | 0.065% |
| Total | 9.86 | 24.3% | 0.616 | 0.56% | 0.231 | 0.52% |
